# The Importance of Biologically Relevant Microclimates in Habitat Suitability Assessments

**DOI:** 10.1371/journal.pone.0104648

**Published:** 2014-08-12

**Authors:** Johanna Varner, M. Denise Dearing

**Affiliations:** Department of Biology, University of Utah, Salt Lake City, Utah, United States of America; Field Museum of Natural History, United States of America

## Abstract

Predicting habitat suitability under climate change is vital to conserving biodiversity. However, current species distribution models rely on coarse scale climate data, whereas fine scale microclimate data may be necessary to assess habitat suitability and generate predictive models. Here, we evaluate disparities between temperature data at the coarse scale from weather stations versus fine-scale data measured in microhabitats required for a climate-sensitive mammal, the American pika (*Ochotona princeps*). We collected two years of temperature data in occupied talus habitats predicted to be suitable (high elevation) and unsuitable (low elevation) by the bioclimatic envelope approach. At low elevations, talus surface and interstitial microclimates drastically differed from ambient temperatures measured on-site and at a nearby weather station. Interstitial talus temperatures were frequently decoupled from high ambient temperatures, resulting in instantaneous disparities of over 30°C between these two measurements. Microhabitat temperatures were also highly heterogeneous, such that temperature measurements within the same patch of talus were not more correlated than measurements at distant patches. An experimental manipulation revealed that vegetation cover may cool the talus surface by up to 10°C during the summer, which may contribute to this spatial heterogeneity. Finally, low elevation microclimates were milder and less variable than typical alpine habitat, suggesting that, counter to species distribution model predictions, these seemingly unsuitable habitats may actually be better refugia for this species under climate change. These results highlight the importance of fine-scale microhabitat data in habitat assessments and underscore the notion that some critical refugia may be counterintuitive.

## Introduction

Anthropogenic climate change has already profoundly affected range and community structure for many taxa [1]–[5]. Predicting future changes in species distributions and interactions is a challenge with great consequences for conserving biodiversity. However, realistic predictions and viable conservation plans depend on accurate forecasts of future habitat suitability.

Species distribution models (SDMs) are the most common approach to predicting habitat suitability under climate change. In this approach, a species’ bioclimatic envelope is statistically determined from its present distribution, and suitable habitats are then predicted where similar climatic conditions will occur in the future [6]. Correlative SDMs have been criticized for failing to incorporate important factors like dispersal, biotic interactions, adaptation and behavioral plasticity [7]. However, the predictive power of these models is improved when used in conjunction with other approaches such as phylogenetics [8], mechanistic heat transfer models [9] or eco-physiological parameters [10].

In spite of recent criticism, few SDMs account for fine scale microhabitat features, which can profoundly influence microclimates and therefore habitat suitability [6], [11], [12]. In fact, a recent meta-analysis demonstrated that average climate grid lengths in SDMs were 10,000-fold larger than the animals they study [13]. In addition, few studies explicitly incorporate data from the periphery of a species’ range, though these populations may be particularly informative of climatic tolerance or critical habitat features [14].

The concept of refugia from climate change has received a great deal of recent attention in the literature [15]–[18]. Refugia are “safe-haven” habitats where species can persist in times of environmental change and may offer hope for *in situ* persistence, particularly for species with poor dispersal capacity [15]. A key feature of refugia is that their microclimates must be relatively stable and must buffer species against climate variability [17]. However, refugia can be difficult to identify, particularly when they are surrounded by areas of less suitable habitat [17]. Indeed, a key aim of conservation science is identifying and protecting habitat features that can serve as refugia through environmental changes.

In this study, we examined how micro-refugia may allow a climate-sensitive species to persist in habitat that appears unsuitable by the bioclimatic envelope approach. American pikas (*Ochotona princeps*) are small mammalian herbivores that have become widely considered indicators of climate change [19]. We hypothesized that vegetation features may create favorable microclimates at low elevations by insulating against summer heat stress, which is known to affect pikas. Uncovering microhabitat variation in seemingly unsuitable climates provides important information about the thermal tolerance, capacity for plasticity and potential vulnerability of a species to future climate change. This information will therefore be vital for refining SDMs and targeting critical habitat refugia for conservation action.

## Materials and Methods

### Study organism

American pikas (*Ochotona princeps;* order *Lagomorpha*) are small mammalian herbivores that are typically distributed in high elevation mountains of western North America because they require short, cool summers and winters with extended snowpack [20]. Pikas are obligate talus specialists and are rarely found outside of rockslides and boulder fields that provide these suitable microclimates. In addition, pikas are extremely sensitive to high ambient temperatures [21], [22] and possess a limited dispersal ability [23], [24]. Unlike most alpine mammals, pikas do not hibernate during the winter but spend the short alpine summer amassing food caches called haypiles [25]. It has been hypothesized that warm summer temperatures may prevent pikas from constructing adequate haypiles, resulting in over-winter mortality [26]. This effect may be compounded by reduced snowpack, which exposes the animals to colder winter temperatures [27]–[29].

With climate change, parts of the pika’s range are becoming unsuitable, resulting in upslope range retractions [27], population declines [28], and localized extinctions [27], [30], [31]. However, pikas persist in some regions with warm climates, including the Columbia River Gorge (CRG) in Oregon and Washington. Little study has been devoted to CRG pikas, though they exist over a thousand meters below the species’ previously recognized bioclimatic envelope [20], [32]. Given this species’ demonstrated sensitivity to aspects of climate, marginal populations like the CRG have great potential to elucidate crucial features of micro-refugia and tractable conservation targets.

### Study sites

We collected microhabitat data at four talus patches (ca. 15,000 m^2^ each; hereafter, “sites”) occupied by pikas in the CRG during June 2012–June 2014 (Table 1, Fig. S1). All sites were 32–35° in steepness, north to northwest facing and surrounded by a dense forest. One of the unique features of this region is a thick layer of moss that covers 25–80% of the surface of each rockslide. The moss also serves as a substrate for other plant cover, including graminoids, forbs and ferns. Sites ranged in elevation from 94 m to 437 m and varied in moss cover from over 65% (“high moss cover”: sites 1 & 2) to less than 30% (“low moss cover”: sites 3 & 4; Table 1, Fig. S1). Each of these sites is about a thousand meters lower in elevation than pikas are predicted to occur, based on the climate envelope approach [32].

**Table 1:** Study sites in the CRG (elev. <500 m) and Mt. Hood (elev. >900 m).

Site	Latitude	Longitude	Elev. (m)	Veg.Cover(%)^a^	MossCover(%)	Avg.ClastSize (cm)	Aspect(deg.)	Slope(deg.)	Insolation^b^
Site 1	45°41′49″N	121°39′10″W	94	75.6	61.0	24.6	342	33	0.52
Site 2	45°41′16″N	121°47′45″W	194	71.2	68.6	40.1	2	32	0.53
Site 3	45°40′25″N	121°50′17″W	281	37.0	35.3	53.0	314	35	0.40
Site 4	45°33′50″N	122°09′14″W	437	35.7	25.5	48.7	313	33	0.37
Site 5	45°27′31″N	121°40′24″W	905	36.2	33.3	50.7	349	27	0.45
Site 6	45°24′41″N	121°41′59″W	1682	9.8	1.1	53.8	36	28	0.38

a Vegetation cover includes moss, ferns, grass, forbs and shrub cover on the talus.

b Insolation = sine(slope) × cosine(aspect), from [33]. See Materials and Methods for interpretation of values.

We estimated potential solar exposure at each site using a previously-described insolation index [33]. The aspect and slope angle of each site were measured using a compass equipped with an inclinometer, and potential solar insolation was calculated as sine(slope) × cosine(aspect). This index ranges from −1 to 1, where values of 1 indicate steeper north-facing slopes with little solar exposure, and values of −1 indicate steep south-facing slopes with high exposure [33].

We also sampled alpine microhabitats typical for pikas at two additional sites on the north face of Mt. Hood, approximately 30 km from sites 1–4 (Table 1). Clast size was similar across CRG and Mt. Hood sites, with most rocks at each talus site having dimensions in the range of 20–100 cm, which is preferred for pikas (Table 1). All six sites were along publicly accessible trails on land owned by the United States Forest Service or Oregon Parks and Recreation Department. Appropriate permits for deploying dataloggers in Oregon State Parks were acquired from Oregon Parks and Recreation Department, permit no. 012-11.

### Macroclimate temperature measurements

We downloaded ambient temperature data of the type used for SDMs from the Western Regional Climate Center (available: http://www.raws.dri.edu/wraws/orF.html) at the Cascades Locks station (45°40′10″N, 121°52′54″W, elevation 128 m). This weather station is centrally located in the CRG, 10.6 km from our four low elevation field sites, on average. It is closest (3.4 km) to site 3. To further characterize ambient temperatures at CRG sites, HOBO Pendant temperature dataloggers (model UA001-08; Onset Computer, Bourne, MA) were suspended from a tree branch approximately 2 m above the ground and protected from solar radiation by a white plastic shield. All measurements labeled “ambient” reflect these 2 m shade air temperatures.

### Microclimate temperature measurements

We measured temperatures in pika-relevant microhabitats with HOBO Pendant temperature dataloggers, which were housed in waterproof, plastic cases. At each site, we placed loggers at the talus surface in vegetated areas near the bottom of the talus slope where we had observed pikas foraging [34]. All surface loggers were shielded from direct solar radiation by moss. To characterize sub-surface temperatures, we threaded a second, paired sensor into the talus interstices to reach a final depth (i.e., vertical distance directly below the surface logger) of 80–100 cm. In some cases, small sub-surface rocks were removed for sensor insertion and immediately replaced, but vegetation cover at the surface was not disturbed. Finally, at sites 2 and 3, we deployed a third logger in the surrounding forest to characterize a potential midday thermal refuge. For consistency, these forest surface loggers were also shielded from solar radiation by moss.

Average summer temperature is known to impact pikas [27]–[29], [33]. Furthermore, summer temperatures in the CRG are particularly unusual for this species [32]. To adequately capture average summer temperatures and to characterize spatial variation in summer temperatures, we deployed three additional surface-interstitial temperature logger pairs at each site during June–August 2013. To control for daily patterns of shade cover on the talus, one logger pair was placed near the top of the talus slope, and the other two logger pairs were placed approximately halfway up the talus on the east and west sides. Thus, at each site, we collected temperature measurements every two hours at four locations at the talus surface and four locations in the talus interstices during this time period. All datalogger locations were placed in areas of the talus where pikas were observed foraging, determined from behavioral observations [34].

Finally, to provide an initial characterization of humidity patterns between sites, we deployed HOBO relative humidity logger (model U12-011; one per site) at sites 2 and 3 during June 2012–August 2013. Loggers were placed in plastic housing with two mesh walls that permitted sufficient air exchange for accurate humidity readings but prevented the logger from direct contact with substrates or precipitation. This housing was then threaded approximately 80 cm into the talus interstices.

### Spatial variation in temperature

To characterize thermal variation at very small scales, we deployed additional surface, interstitial and ambient loggers at site 2 and site 3 from July 14–August 30, 2013 in close proximity (1 m) to the sensor network described above. We then characterized spatial variation in temperatures within and between sites with variograms, which are widely used in geostatistics to represent autocorrelation between measurements observed at different spatial locations. Variograms were recently recommended as a spatially explicit tool for examining thermal variance in microclimates as a function of distance between measurements [13]. The semivariance (γ) of temperature measurements at a given time point is half the average squared difference between logger-values (*x*), separated by a distance *h*, as given by: 
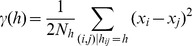
, where *i, j* are specific logger pairs separated by distance *h,* and *N_h_* is the number of logger pairs that are separated by this distance [35]. Lower semivariance therefore indicates higher autocorrelation between temperature measurements at a given separation distance. We computed semivariances with the *variogram* function in the R package *gstat* [36], [37] for temperature measurements during June–August 2013. This function calculated semivariance at four average separation distances: 1 m (between closely placed loggers at sites 2 and 3), 39 m (between loggers within a site), 3,622 m (between loggers at nearby sites) and 10,908 m (between loggers at distant sites).

### Vegetation cover and microclimate

To provide an initial test of the hypothesis that vegetation insulates against extreme surface temperatures, we manipulated vegetation cover at an additional talus patch (ca. 1 km from site 1). A patch of moss approximately 1 m in diameter was experimentally removed and relocated nearby where moss did not naturally grow (Fig. S2). We then measured temperatures every two hours from May 28–June 13, 2012 at four locations: under an unmanipulated patch of moss, under the transplanted moss, under a pile of bare control rocks that did not naturally have any moss, and under the pile of bare rocks where we removed moss (Fig. S2). The bare rocks in this experiment were approximately 15–20 cm in average dimension. All four locations were within 2 m of each other, and therefore should experience similar patterns of shade and sun exposure throughout the day. We compared daily average and maximum temperatures with two-way, repeated measures ANOVA, using treatment (i.e., manipulation vs. control) and cover (i.e., moss vs. rocks) as main effects and date as a repeated measure.

### Microclimate and elevation

Temperature should decrease as elevation increases according to region-specific lapse rates, which may also vary by season [38]. In this region, previously reported lapse rates are −2°C/m for minimum temperatures and −7°C/m for maximum temperatures [39]. To investigate the impact of elevation on microclimate and calculate pika-relevant lapse rates, we placed paired surface-interstitial dataloggers at each high elevation site, which recorded temperatures at the same two-hour intervals as CRG loggers during June–August 2013. To test the effect of elevation on temperatures in pika-relevant microclimates, we computed Pearson correlation coefficients between elevation and average, maximum and minimum temperatures when all sites were free of snow.

### Data deposition

The temperature and relative humidity measurements collected for this study are freely available in USPACE (http://uspace.utah.edu), the University of Utah’s institutional repository. The accession number for these data is ark:/87278/s6b583mp (available: http://content.lib.utah.edu/cdm/ref/collection/uspace/id/10610).

## Results

### Macroclimate temperature measurements

We found no difference between 2 m shade ambient temperatures collected at sites 1–3 and temperatures measured at the Locks weather station for daily averages (*F_3,1288_* = 0.654, *p* = NS), maxima (*F_3,1288_* = 2.162, *p* = NS) or minima (*F_3,1288_* = 0.654, *p* = NS) collected between October 2012 and August 2013. We therefore present data from our ambient loggers in future analyses because these data were collected at the same two-hour intervals as microclimate data.

### Microclimate temperature measurements

**Figure 1:**
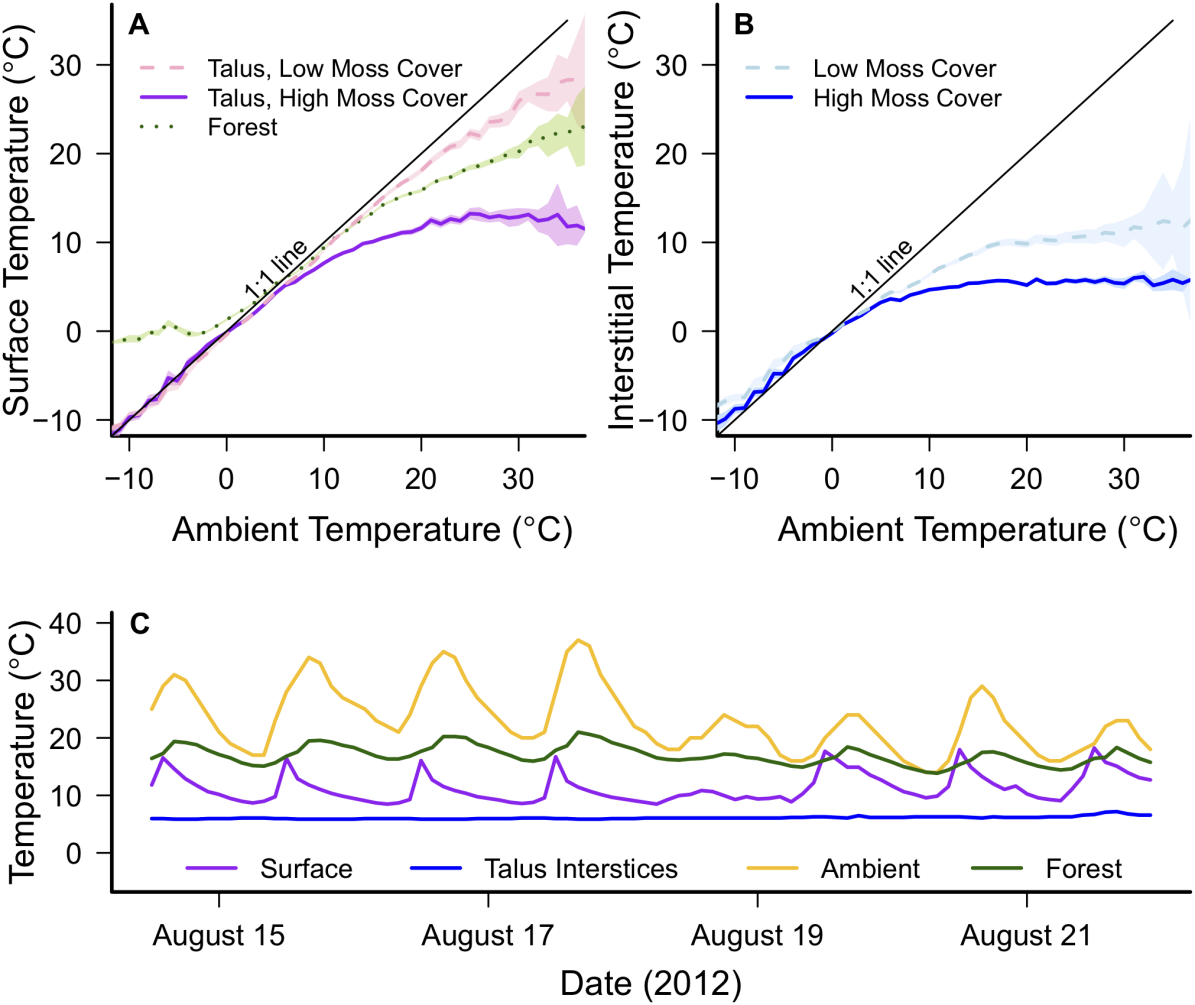
Pika microclimates compared to ambient temperatures. Temperature data were collected every two hours from June 2012 to June 2014 at four sites in the Columbia River Gorge. (A) Lines represent mean temperatures and shaded areas represent 95% confidence intervals. At high ambient temperatures, talus surface temperatures were lowest at sites with high moss cover. Forest surface temperatures were also cooler than ambient temperatures, but talus surface temperatures at sites with low moss cover closely tracked ambient temperatures. (B) Talus interstitial temperatures were functionally decoupled from variation in ambient temperature and remained a cool and constant 4–7°C at sites with high moss cover. (C) Time-trace of ambient, talus surface, forest surface, and talus interstitial temperatures measured at site 2 (high moss cover) during August 2012.

We observed substantial divergence in microclimates relevant to pikas compared to 2 m shade ambient temperatures (Fig. 1). Although surface sensors were below moss cover at all sites, patterns in talus surface temperature were dependent upon overall moss cover at the site. At sites with high moss cover (>65% coverage), talus surface temperatures rarely exceeded 20°C, even at ambient temperatures over 35°C (Fig. 1A, C). In contrast, at sites with low moss cover (≤35% coverage), talus surface temperatures were only a few degrees cooler than ambient temperatures, on average (Fig. 1A). Temperature at the forest floor was also 4–9°C cooler at high ambient temperatures, but rarely dipped below 0°C on very cold days (Fig. 1A).

At all sites, interstitial temperatures were strongly divergent from ambient temperatures (Fig. 1B). Even at ambient temperatures over 35°C, interstitial temperatures at sites with high moss cover remained a cool and constant 6–8°C, resulting in instantaneous disparities of up to 31.5°C between 2 m shade ambient temperatures (measured on-site) and microhabitats relevant to pikas (Fig. 1C). Daily variation in interstitial temperature was <1°C during the summer at sites with high moss cover, essentially decoupling interstitial temperatures from temperatures at the surface (Fig. 1B, C). At sites with low moss cover, interstitial temperatures were more variable but remained 5–11°C cooler on average than the talus surface.

Finally, relative humidity varied with ambient temperature (Fig. S3). At ambient temperatures below 10°C, both sites 2 and 3 had uniformly high relative humidity (>90%). At warmer ambient temperatures, relative humidity at both sites became more variable. However, site 2 (high moss cover) had consistently higher relative humidity (75–95%) than site 3 (low moss cover; 55–75% humidity).

### Spatial variation in temperature

**Figure 2:**
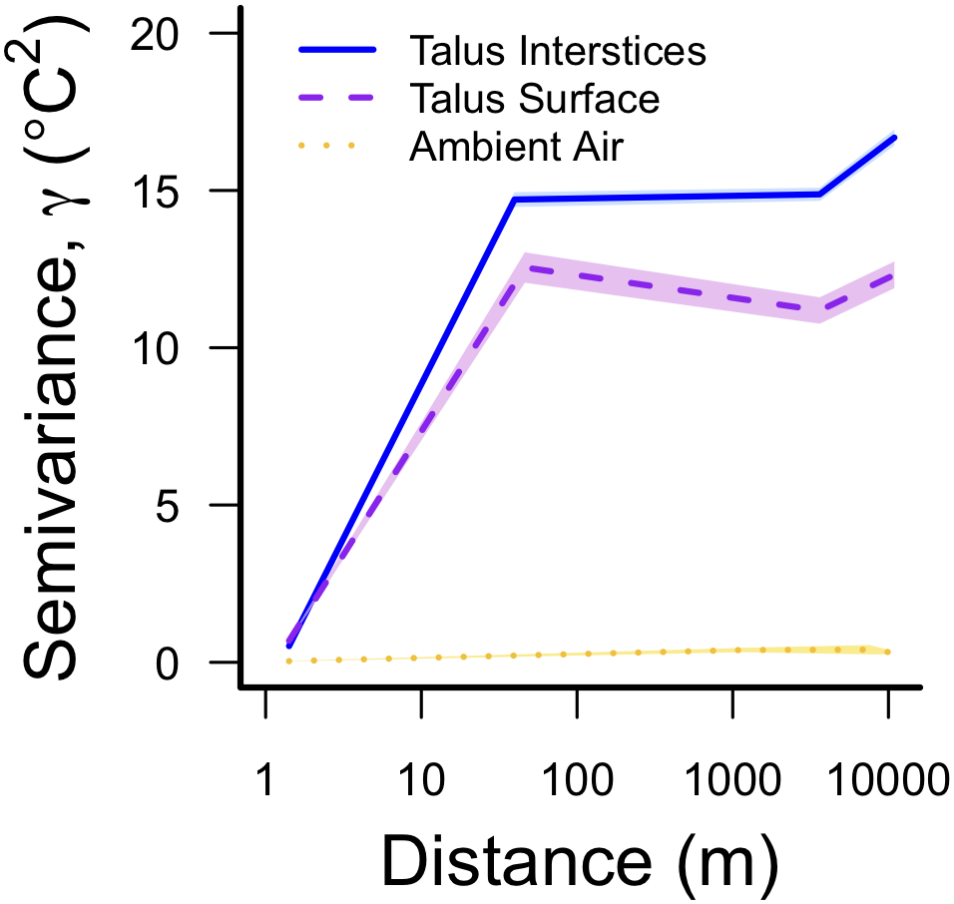
Spatial variation in pika-relevant microclimates and ambient temperatures. Thermal semivariance is shown as a function of distance between dataloggers at the 4 CRG sites during June–August 2013. Lines represent mean semivariance and shaded areas represent 95% confidence intervals. Temperatures in pika-relevant microclimates are far more heterogeneous and less spatially correlated than ambient shade temperatures measured 2 m above the ground.

Semivariance, or the average squared difference in temperature measurements separated by a certain distance, remained very low (<0.4°C^2^) among ambient temperatures, indicating high autocorrelation between these measurements at all scales (Fig. 2). In contrast, spatial variation in pika-relevant microclimates far exceeded spatial variation in ambient temperatures at all scales (Fig. 2). At a 1 m separation distance, semivariances for temperatures recorded at the talus surface and in talus interstices were relatively low (<1°C^2^), indicating that temperature measurements separated by 1 m were highly correlated. However, at larger spatial scales, semivariance quickly increased to 12°C^2^ for temperatures recorded at the talus surface and 16°C^2^ for temperatures recorded in the talus interstices, indicating a very low degree of spatial autocorrelation. At separation distances larger than 1 m, microclimate temperatures recorded within the same site were not more similar to each other than temperatures recorded by sensors at the most distant sites.

### Vegetation cover and microclimate

**Figure 3:**
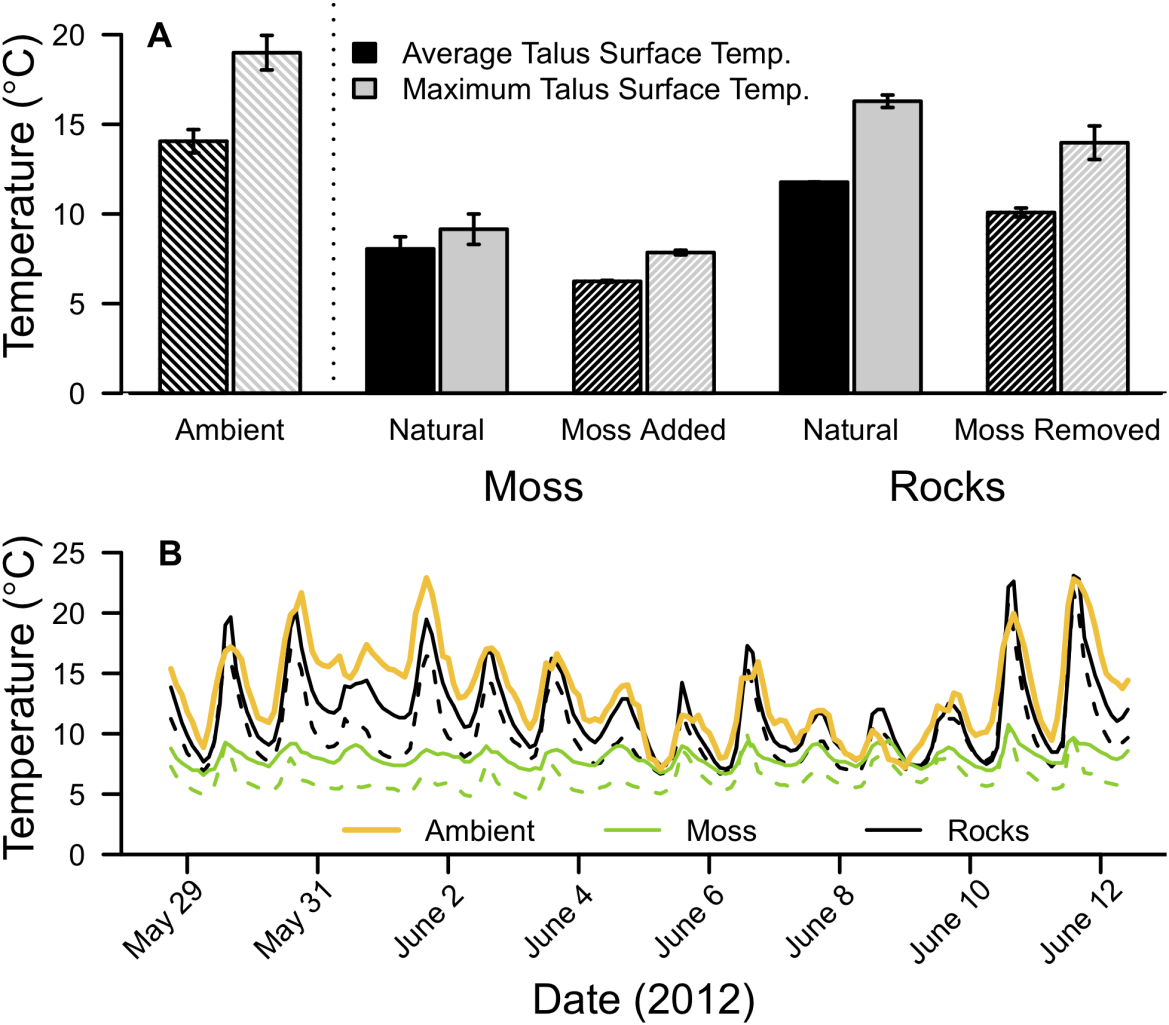
The effect of vegetation cover on summer surface temperatures. (A) Average and maximum summer surface temperatures were significantly cooler under moss, compared to rocks. Both types of surface temperature were cooler than ambient temperatures. (B) Time-trace of temperatures during this experiment.

During the summer, average talus surface temperatures were 5–9°C cooler under both naturally occurring and transplanted moss, compared to bare rocks (Cover: *F_(1,31)_* = 239.2, *p*<0.0001; Fig. 3A). Surprisingly, there was also a significant effect of treatment, such that unmanipulated moss and rocks were also 1–2°C warmer than manipulated moss and rocks (Treatment: *F_(1,15)_* = 118.6, *p*<0.001), but there was no interaction between cover and treatment. Maximum surface temperatures exhibited the same pattern (Cover: *F_(1,31)_* = 131.1, *p*<0.0001; Treatment: *F_(1,15)_* = 79.1, *p*<0.001; Fig. 3A). Over the duration of this experiment, both types of surface (moss and rocks) were cooler, on average, than ambient air temperature (Fig. 3A). However, surface measurements under rocks more closely tracked ambient temperatures; whereas surface measurements under moss were often up to 15°C cooler than ambient air temperature (Fig. 3B).

### Microclimate and elevation

**Figure 4:**
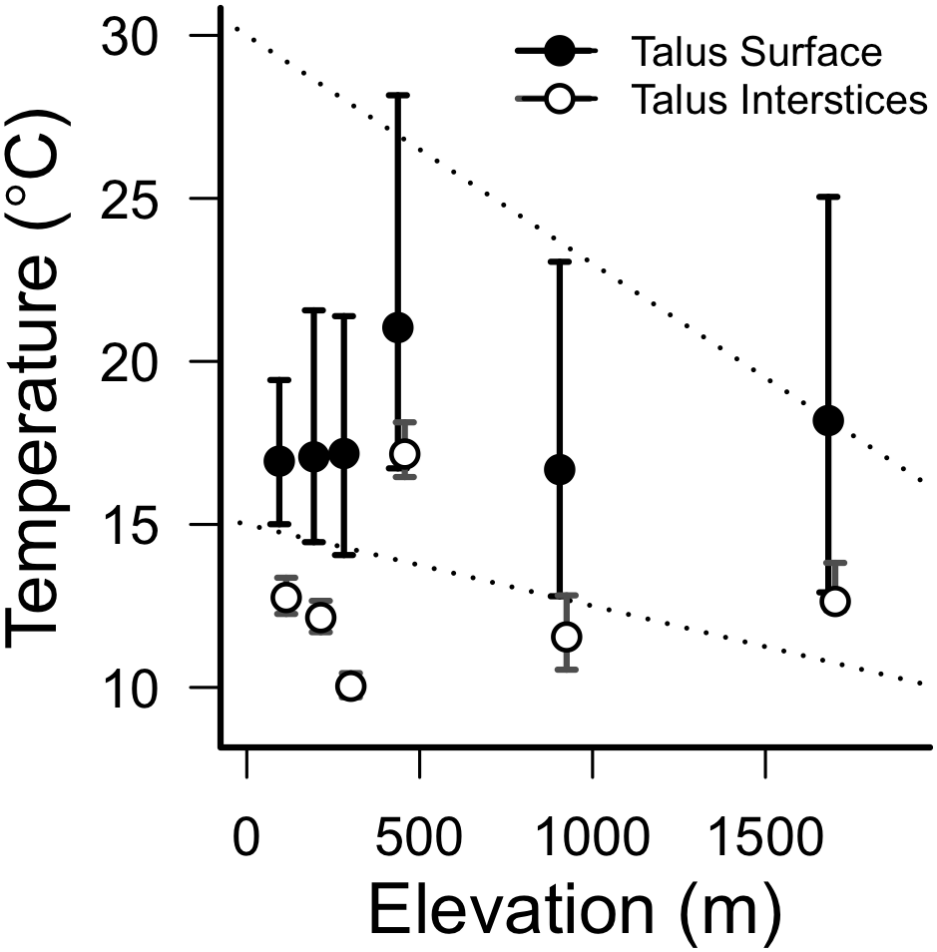
Average summer temperatures by elevation. Points represent mean summer temperatures at the talus surface (filled) and 1 m into talus interstices (open). Bars range from mean daily maxima to mean daily minima in each of these habitats. To show bars that overlap, interstitial points are shifted right by 20 m at some sites. Dotted lines represent previously-calculated lapse rates for the Pacific Northwest, from [39].

Summer temperatures in the CRG were substantially cooler than predicted by region-specific lapse rates ([39]; Fig. 4). In fact, elevation was not predictive of talus interstitial temperatures or surface temperatures. All Pearson correlation coefficients between elevation and average, minimum or maximum temperatures were ≤0.6 and non-significant (*p*>0.2). The lowest elevation sites also had less than half the daily temperature range (i.e., daily maximum – daily minimum) of high elevation sites.

## Discussion

We investigated the effects of vegetation and elevation on microclimates relevant to a sensitive species living in an atypical habitat. On the basis of climate envelope models alone, the Columbia River Gorge (CRG) appears to be unsuitable habitat for pikas because it does not have short, cool summers or long winters, which are commonly recognized as criteria for pika persistence [e.g., 20,32,40]. However, our results demonstrate that cool microclimates are available in the CRG and that these low elevation rockslides may actually be more suitable for pikas than the nearby alpine talus that is more typical for this species.

Interstitial temperatures in the talus typically depend on heat-transfer processes at the surface (i.e., convection, conduction, solar and long-wave thermal radiation). However, during the summer in the CRG, daily fluctuations in this microhabitat are frequently less than 0.2°C and instantaneous disparities with ambient temperatures may be up to 31.5°C (Fig. 1C), suggesting that, at some locations, the surface heat-transfer processes do not penetrate more than 1 m into the talus. As a result, interstitial microclimates are functionally decoupled from heat-transfer at the surface and are likely governed by other processes. CRG taluses have several features that are atypical for pika habitat, including a thick layer of vegetation covering the talus, high relative humidity, and reduced solar insolation (i.e., due to slope orientation and/or shade from the surrounding forest canopy). Each of these features may contribute to the significant disparities observed between the microclimate relevant to pikas and the macroclimate observed at weather stations.

Vegetation features, and moss cover in particular, appear to buffer against climate variability, differentiating talus microclimates at some sites from the surrounding macroclimate (Fig. 1). Our high moss cover sites likely experience more shade (Table 1) and higher humidity (Fig. S3). These features may facilitate moss growth because mosses are predisposed to grow in shady, humid conditions [41]. However, our experimental results suggest that mosses may also actively buffer talus microclimates from ambient temperature extremes (Fig. 3). In other ecosystems and urban environments, vegetation can similarly lower surface temperatures of dark substrates by 5–20°C [42], . This cooling effect may be caused by changes in diffuse reflectivity (albedo), which can strongly affect surface temperatures. Specifically, mosses may raise the albedo of the dark basalt that makes up the talus in this region, increasing the amount of solar radiation that is reflected. The albedo of basalt is 0.1, whereas albedo estimates of moss and lichen-dominated surfaces are two to four times higher [0.2–0.4; 44]. For comparison, a change of albedo in 0.2 is capable of producing a 10°C change in midday surface temperatures [45]. In addition to increasing albedo, vegetation cover may also act as a layer of insulation [44], or it may have an evaporative cooling effect.

Relative humidity and/or precipitation may also play an important role in pika persistence and abundance in this habitat by maintaining high quality vegetation or water for evaporative cooling. During our study, the Locks weather station near our sites averaged ∼150 mm of annual rainfall and 70–80% relative humidity. Similarly, relative humidity in the talus rarely dipped below 75% at our high moss cover sites (Fig. S3). Although differences in relative humidity may be responsible for the distribution of vegetation cover on talus in this region, the vegetation itself may also locally raise humidity through evapotranspiration. Although we did not explicitly measure their effects on microclimates, sub-surface water or ice may also influence microclimate temperatures in this region. Water availability is a strong determinant of pika persistence and abundance in other parts of their range [30], [46], and we regularly experienced cold air flowing out of talus cavities, which may indicate subsurface rock-ice or water features [47].

These low elevation sites also experience relatively little solar radiation due to northerly slope orientations and daytime shade cover provided by the surrounding forest canopy. This reduced insolation likely has a large effect on both the magnitude and variability of temperatures in pika habitat. For example, although we did not directly measure solar radiation during this study, site 4 likely received more sunlight because its surrounding forest canopy is shorter than other sites due to a wildfire in 1991 (Fig. S1). Indeed, although they are similar in elevation, slope angle, aspect and moss cover, summer surface and interstitial temperatures were approximately 5°C warmer and much more variable at site 4, compared to site 3 (Fig. 4). Similarly, a shorter forest canopy and higher insolation indices at our high elevation sites may have also contributed to greater temperature variability, compared to the CRG (Fig. 4). Unlike high elevations, CRG microclimates rarely experienced temperature extremes beyond the best estimates of thresholds for acute heat stress (25.5°C) [22] or cold stress (−10°C) [27] in this species.

Shade and vegetation cover are also likely responsible for the mild surface temperatures that we observed in the forest at sites 2 and 3 (Fig. 1A). Consistent with our findings, forest surface temperatures in other studies are typically 5–8°C lower than ambient temperatures measured above the ground [48], [49]. Forest surface temperatures were typically a few degrees warmer than talus surface temperatures at sites with high moss cover (Fig. 1A, C), but the forest may represent an important thermal refuge for pikas to remain active at midday at sites with low moss cover. Similarly, the forest may also serve as a winter refuge for CRG pikas, as forest temperatures also remained warmer than talus temperatures on the few very cold days in 2012–2014 (<−10°C; Fig. 1A). Finally, thick understory vegetation in the forest may also make pikas less visible to predators or serve as an additional food resource [34].

Regardless of mechanism, the temperature disparities that we observed between weather stations and talus interstices in the CRG far exceed those in the microclimate literature. Previous studies examining ambient temperatures and microclimates at or below the soil surface report disparities up to 10°C between these measurements [12]. Similarly, burrow systems have a significant ability to buffer animals against temperature fluctuations, but instantaneous differences between ambient shade temperatures and internal burrow temperatures rarely exceed 10°C in the literature [50]–[55]. Rock-ice features in pika habitat in the Sierra Nevada range can also cool the average warm-season temperatures in talus interstices by 3.8°C, compared with the surface. In contrast, we observed differences of up to 11°C between average surface and interstitial microclimates in the warm season, and instantaneous disparities up to 31.5°C between talus interstitial temperatures and ambient temperature measured on site. For comparison, this disparity is roughly equivalent to 4500 m of elevation change (according to lapse rates for this region) and far exceeds the magnitude of observed and predicted directional climate change.

CRG microhabitats were also highly heterogeneous. A lack of spatial autocorrelation between temperature measurements (even between loggers at the same site, Fig. 2) suggests that spatially uncorrelated noise exceeds deterministic trends in temperature at scales over ∼10 m in these microhabitats. This result may be due to the patchy nature of moss and vegetation cover on the talus or to the movement of shade cover across the talus throughout the day. Interestingly, strong correlations were observed in the Great Basin between interstitial temperatures in montane pika habitat and ambient temperatures measured at valley weather stations 40–60 km away [29]. The temperature disparities and spatial heterogeneity that we report here highlight the uniqueness of habitats with high vegetation cover, and more generally, the refugial value of places where surface conditions can partially decouple ambient temperatures from sub-surface microclimates. Importantly, high spatial heterogeneity in a habitat may also allow animals to behaviorally mitigate the impacts of directional climate change by shuttling between different areas within a habitat [12].

Taken together, these results suggest that the low elevation habitat in the CRG may actually be a better refuge from stresses caused by climate change than typical, high elevation habitat and that CRG pika populations have the potential to be relatively resilient to future environmental change. These results are a notable example of an unexpected or counter-intuitive thermal refuge, since the CRG was predicted to be unsuitable based on SDM results alone [e.g., 20,32,40]. Our results also suggest tractable conservation priorities for pikas and other thermally sensitive species in this region. Specifically, land managers could focus on protecting habitat features that contribute to the unique microclimates, including moss (e.g., from trampling by hikers or unsustainable harvest for the horticulture trade) and forest canopy cover (e.g., from logging or severe wildfires).

An ecologically similar species that is likely to be directly affected by talus microclimates in this region is the bushy-tailed woodrat, *Neotoma cinerea*. Like pikas, these woodrats inhabit exclusively rocky habitats in montane forests and are sensitive to high ambient temperatures [56]. This species has experienced localized extinctions in response to past climate change [57] and appears to be suffering a range collapse in response to contemporary climate change [1]. However, we have observed fresh woodrat sign at many talus patches in the CRG and speculate that cool microclimates and high vegetation availability also facilitate woodrat persistence at this unusually low elevation.

Course-scale SDMs should not be discarded as a predictive tool, but climate data and their resolution must be selected with great care. We echo calls from Potter et al. [13] that the ideal spatial resolution for climate data in SDMs must be related to the body size of the species of interest. However, we also suggest that heterogeneity of microclimate features and the spatial or temporal extent to which an organism samples these features should also inform selection of appropriate climate data in SDMs. For example, larger, less-mobile organisms are more likely to experience macroclimates observed at greater spatial grid lengths. Conversely, our results demonstrate that ambient temperature data collected at distant locations may have relatively little relevance for small, mobile species that can rapidly shuttle between complex microhabitats, particularly in environments or seasons when surface features such as snow or vegetation decouple macro- and microclimates. Finally, the timing, frequency and amount of precipitation are rarely part of habitat suitability analyses, though they should be considered.

Microhabitat data will be critical for developing and validating spatial statistics to understand the relationship between climate change and microclimate stability [13]. Relatively little is known about the degree to which microclimates will respond to macroclimatic changes or the time-scale on which these changes will occur. Collecting organism-relevant microhabitat data at finer spatial resolutions and longer time scales will be critical for bridging this gap and for determining the resiliency of microclimate refugia to continued changes in broad-scale temperature and precipitation patterns. Such studies are becoming increasingly feasible, given the availability of relatively inexpensive and small temperature dataloggers with long battery lives.

Our results highlight the need for fine-scale temperature measurements in microhabitats that are relevant to a focal species. When identifying refugia from climate change for conservation action, we can learn much from fine-scale observations of populations in seemingly marginal climates. Indeed, the conservation value of refugia lies in promoting species’ ability to persist even under unfavorable climates [16].

## Supporting Information

Figure S1 **Comparison of moss cover at four sites in the Columbia River Gorge.** For reference, pikas are also shown at sites of high (E) and low moss cover (F). *Photo credits*: (A–E) J. Varner, (F) J. J. Horns.
(TIFF)

Figure S2 **Moss transplant experiment.** (A) In an area of the site where moss did not naturally grow, temperatures were measured under rocks and transplanted moss. (B) In an area of the site naturally covered with moss, temperatures were measured under rocks where moss was removed and a patch of unmanipulated moss. Arrows indicate datalogger locations.
(TIFF)

Figure S3 **Relative humidity compared to ambient temperature.** Relative humidity measurements were collected every 2 hours in the talus interstices at sites 2 and 3 during June 2012–August 2013. Ambient temperatures (2 m height, shaded) were collected at site 3 at the same temperature intervals. Lines represent mean temperatures and shaded areas represent 95% confidence intervals.
(TIFF)
