# Supplementary figures and images for: The Importance of Biologically Relevant Microclimates in Habitat Suitability Assessments
Source: PLoS One. 2014 Aug 12;9(8):e104648. doi: 10.1371/journal.pone.0104648 (PMC4130583; doi:10.1371/journal.pone.0104648)

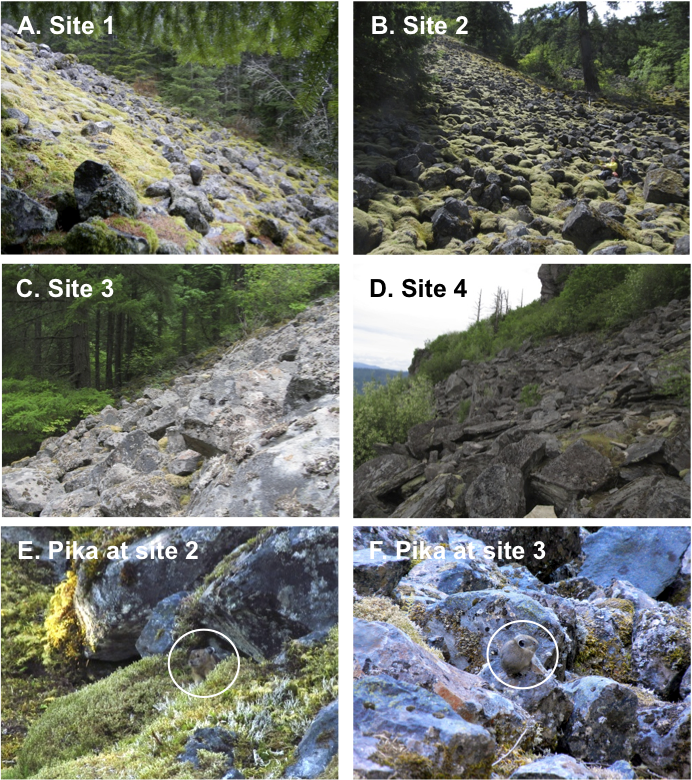

Supplement: Figure S1 — Comparison of moss cover at four sites in the Columbia River Gorge. For reference, pikas are also shown at sites of high (E) and low moss cover (F). Photo credits: (A–E) J. Varner, (F) J. J. Horns. (TIFF) [file pone.0104648.s001.tiff]

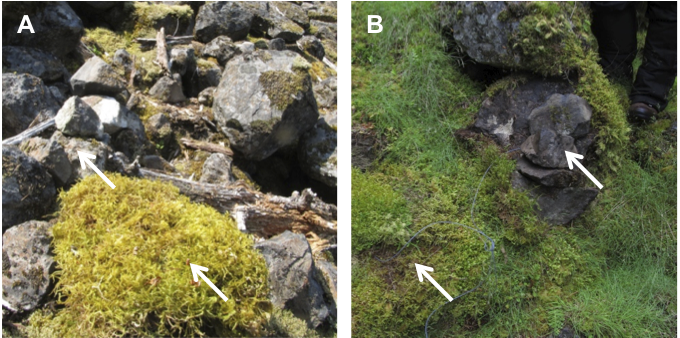

Supplement: Figure S2 — Moss transplant experiment. (A) In an area of the site where moss did not naturally grow, temperatures were measured under rocks and transplanted moss. (B) In an area of the site naturally covered with moss, temperatures were measured under rocks where moss was removed and a patch of unmanipulated moss. Arrows indicate datalogger locations. (TIFF) [file pone.0104648.s002.tiff]

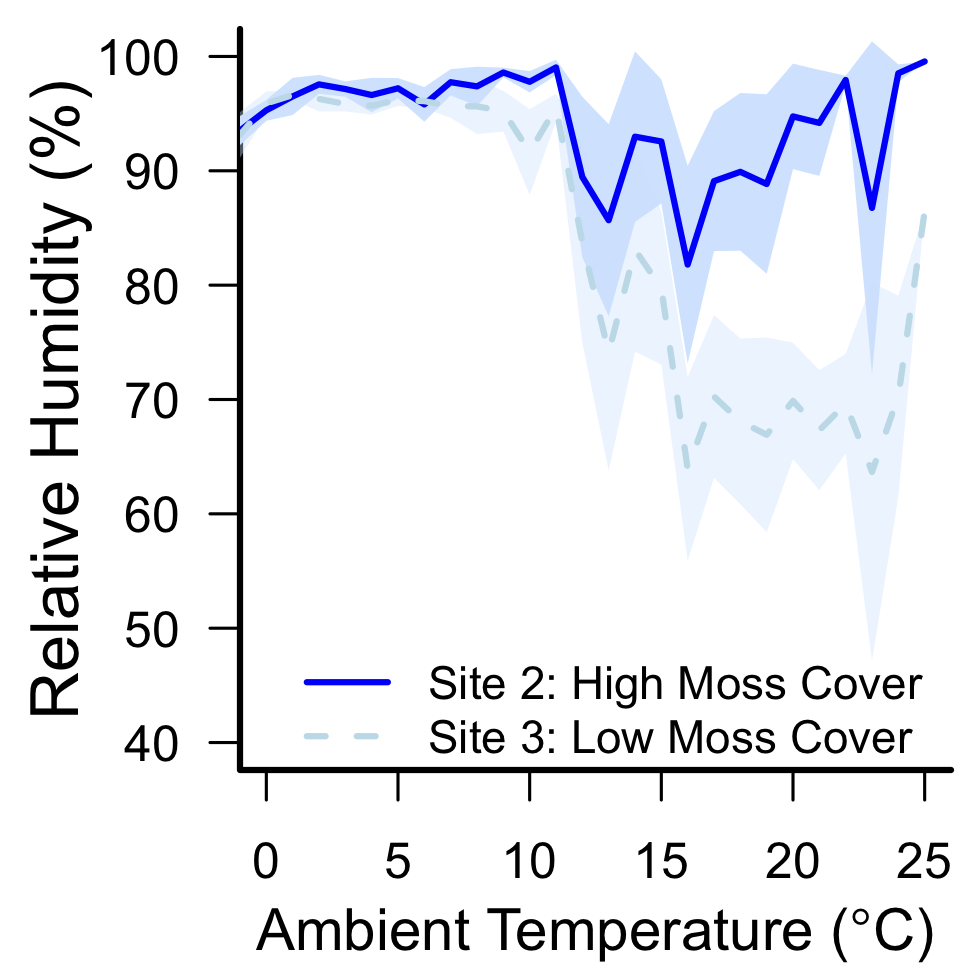

Supplement: Figure S3 — Relative humidity compared to ambient temperature. Relative humidity measurements were collected every 2 hours in the talus interstices at sites 2 and 3 during June 2012–August 2013. Ambient temperatures (2 m height, shaded) were collected at site 3 at the same temperature intervals. Lines represent mean temperatures and shaded areas represent 95% confidence intervals. (TIFF) [file pone.0104648.s003.tiff]
